# Bisphenol A Exposure and Cardiac Electrical Conduction in Excised Rat Hearts

**DOI:** 10.1289/ehp.1206157

**Published:** 2014-01-31

**Authors:** Nikki Gillum Posnack, Rafael Jaimes, Huda Asfour, Luther M. Swift, Anastasia M. Wengrowski, Narine Sarvazyan, Matthew W. Kay

**Affiliations:** 1 Department of Pharmacology & Physiology, and; 2 Department of Electrical and Computer Engineering, The George Washington University, Washington, DC, USA; * These authors contributed equally to this work.

## Abstract

Background: Bisphenol A (BPA) is used to produce polycarbonate plastics and epoxy resins that are widely used in everyday products, such as food and beverage containers, toys, and medical devices. Human biomonitoring studies have suggested that a large proportion of the population may be exposed to BPA. Recent epidemiological studies have reported correlations between increased urinary BPA concentrations and cardiovascular disease, yet the direct effects of BPA on the heart are unknown.

Objectives: The goal of our study was to measure the effect of BPA (0.1–100 μM) on cardiac impulse propagation *ex vivo* using excised whole hearts from adult female rats.

Methods: We measured atrial and ventricular activation times during sinus and paced rhythms using epicardial electrodes and optical mapping of transmembrane potential in excised rat hearts exposed to BPA via perfusate media. Atrioventricular activation intervals and epicardial conduction velocities were computed using recorded activation times.

Results: Cardiac BPA exposure resulted in prolonged PR segment and decreased epicardial conduction velocity (0.1–100 μM BPA), prolonged action potential duration (1–100 μM BPA), and delayed atrioventricular conduction (10–100 μM BPA). These effects were observed after acute exposure (≤ 15 min), underscoring the potential detrimental effects of continuous BPA exposure. The highest BPA concentration used (100 μM) resulted in prolonged QRS intervals and dropped ventricular beats, and eventually resulted in complete heart block.

Conclusions: Our results show that acute BPA exposure slowed electrical conduction in excised hearts from female rats. These findings emphasize the importance of examining BPA’s effect on heart electrophysiology and determining whether chronic *in vivo* exposure can cause or exacerbate conduction abnormalities in patients with preexisting heart conditions and in other high-risk populations.

Citation: Posnack NG, Jaimes R III, Asfour H, Swift LM, Wengrowski AM, Sarvazyan N, Kay MW. 2014. Bisphenol A exposure and cardiac electrical conduction in excised rat hearts. Environ Health Perspect 122:384–390; http://dx.doi.org/10.1289/ehp.1206157

## Introduction

Bisphenol A (BPA) is one of the most widely used chemicals worldwide, with > 8 million pounds produced each year (Vandenberg et al. 2010). BPA is a component of polycarbonate plastics and epoxy resins, which are used in many plastic consumer products, including food and drink containers, water pipes, thermal paper and paper products (e.g., receipts, paper towels), toys, safety equipment, electronics, dental monomers, and medical equipment and tubing. Vandenberg et al. (2007) reported that BPA can leach from these products under normal conditions. Despite the increasing popularity of BPA-free plastics, BPA is still found in many consumer products. Indeed, human biomonitoring studies suggest that a large proportion of the population may be exposed to BPA, including both children and adults (Calafat et al. 2005, 2008). BPA exposure rates range dramatically depending on lifestyle factors, with neonates in intensive care units (4.4 nM–4 μM) and industrial workers (0.024–8.5 μM) having overall higher urinary BPA concentrations (Calafat et al. 2009; Wang et al. 2012). Human serum BPA levels are actively debated, with estimates ranging from 0.001 to 0.3 μM for adults (Kaddar et al. 2009; Lee et al. 2008; Padmanabhan et al. 2008; Schönfelder et al. 2002; Vandenberg et al. 2010). Patients undergoing multiple medical interventions, such as neonates in intensive care, may have even higher levels of BPA in the blood. However, BPA serum levels have not been measured in this patient population.

There is evidence that increased exposure to endocrine disruptors such as BPA might contribute to the onset and progression of disease (Braun and Hauser 2011; Chapin et al. 2008; Diamanti-Kandarakis et al. 2009; Richter et al. 2007; Vandenberg et al. 2007; vom Saal et al. 2007). Recent epidemiological studies have shown an association between BPA exposure and cardiovascular disease. For example, higher urinary concentrations of BPA have been associated with an increased risk of coronary artery disease (Melzer et al. 2010, 2012), hypertension (Shankar et al. 2012), carotid atherosclerosis (Lind and Lind 2011), angina and myocardial infarction (Lang et al. 2008), and a decrease in heart rate variability (Bae et al. 2012). Although the latter is primarily attributed to neuronal influences, decreased heart rate variability can also indicate alterations in ion channel currents that drive pacemaker depolarization (Papaioannou et al. 2013). In addition, *in vitro* experimental studies have shown that higher BPA exposure concentrations modify heart rate in isolated atrial preparations (Pant et al. 2011). Both of these observations suggest alterations of ionic currents in nodal cells, which can influence nodal and bundle branch conduction. Moreover, if BPA induces ion channel alterations in atrial tissue, exposure will likely affect ion channels in other tissue compartments. Importantly, the effect of BPA on heart electrophysiological function has not been examined. We aimed to systematically study the effect of BPA on cardiac conduction by conducting controlled experiments on excised whole rat hearts. These experiments provide new measurements for important electrophysiological parameters, including atrioventricular (AV) conduction and ventricular conduction velocity.

## Materials and Methods

*Animals*. Experiments were conducted using excised hearts from adult female Sprague-Dawley rats (2–3 months of age; body weight, 200–300 g; *n* = 12), purchased from Hilltop Lab Animals (Scottsdale, PA). Studies were limited to females because previous reports (Belcher et al. 2012; Yan et al. 2011) have claimed that the cardiac effects of BPA are sex specific due to its estrogenic properties. To account for the possible effects of estrous cyclicity on cardiac electrophysiology, each animal served as its own control, with all BPA measurements computed as a percent change from the control (no BPA). Animals were housed in The George Washington University (GWU) animal care facility under standard environmental conditions [12:12 hr light:dark cycle, 64–79°C, 30–70% humidity, corn cob bedding (Harlan Laboratories, Indianapolis, IN)] with free access to food (2018 Teklad Global rodent chow; Harlan Laboratories) and carbon-filtered tap water. Animals were housed in groups of 2 or 3 animals per cage for 2 weeks prior to experimentation. All animals were treated humanely and with regard for alleviation of suffering. All procedures were conducted in accordance with the guidelines of the Institutional Animal Care and Use Committee at GWU.

**Figure 1:**
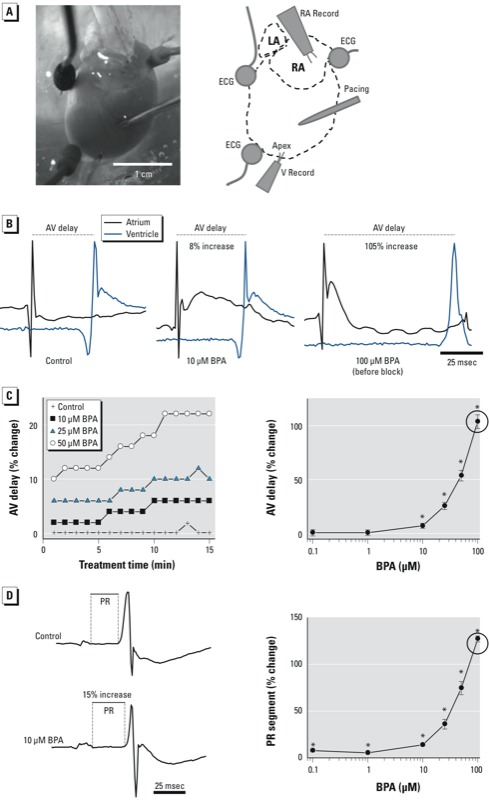
BPA exposure and prolonged AV conduction and ECG segments in excised rat hearts. (*A*) Heart preparation and electrode placement. Abbreviations: LA, left atrium; RA, right atrium; RA Record, right atrium recording electrode; V Record, ventricle recording electrode. (*B*) AV conduction delay after exposure to control media or BPA at 10 or 100 μM. (*C*) AV delay after BPA exposure over time (left), and by dose (0.1–100 μM) measured 15 min after exposure (right). (*D*) PR segment elongation after exposure to 10 μM BPA (left), and PR segment prolongation measured 15 min after exposure to BPA (0.1–100 μM; right). Measurements for 100 μM BPA were performed prior to the onset of AV block (indicated by circle). Values shown in (*C,D*) are mean ± SE; *n* ≥ 3 for each measurement. *p < 0.05. *p*‑Values for dose response were determined by one-way ANOVA; the lowest dose with statistical significance was determined by paired t-tests.

*Excised heart preparation*. Hearts were excised and Langendorff-perfused with a modified Krebs Henseleit buffer (Gillis et al. 1996), as previously described (Mercader et al. 2012). Hearts were placed in a temperature-controlled chamber (Figure 1A) for electrical measurements. To reduce motion, the perfusate was supplemented with 10 μM blebbistatin (Sigma-Aldrich, St. Louis, MO).

*General protocol*. BPA (> 99% purity; Sigma-Aldrich) stock solution was prepared fresh for each experiment. For the stock solution, BPA was dissolved in 100% ethanol. The final dilutions were prepared by adding stock solution directly to the perfusate media to obtain BPA concentrations of 0 (control) to 100 μM, with total ethanol concentrations ranging from 0.01% for the control to 0.02% for 100 μM BPA. Precautions were maintained throughout the study to prevent BPA contamination from external sources [i.e., solutions mixed and stored in glass bottles, or in BPA-free Tygon and C-Flex tubing (Cole Parmer, Vernon Hills, IL) used for Langendorff-perfusion]. However, we did not analyze the perfusate media for BPA contamination.

Excised hearts maintained electrophysiological function for > 3 hr with control media perfusion (no BPA). Each study began midday and was completed in 1–2 hr. The procedure included the following steps: *a*) The excised heart was perfused with control media; *b*) electrodes and electrocardiogram (ECG) leads were positioned (5–10 min); *c*) ECG and AV conduction signals during sinus rhythm were recorded (15-min equilibration period); *d*) the pacing protocol was implemented and optical signals were acquired; *e*) BPA was diluted directly in perfusate media to achieve the appropriate final concentration; *f* ) ECG and AV conduction signals during sinus rhythm were recorded (15 min); and *g*) the pacing protocol was implemented and optical signals acquired. Steps *e–g* were repeated for each BPA concentration.

For each preparation, the heart was exposed to an average of three BPA concentrations added incrementally to the perfusate media (i.e., animal 1: control media → 0.1 μM → 1 μM → 10 μM BPA; animal 2: control media → 1 μM → 10 μM → 25 μM BPA); each exposure lasted 15 min. Each animal served as its own control, with measurements normalized to control perfusion in the same heart and expressed as a percent change. This allowed us to account for variations in electrode position for each experiment and to reduce any influence of estrous cyclicity on cardiac conduction measurements.

The epicardial pacing protocol consisted of pacing the heart with 2 mA pulses (5 msec duration) for ≥ 5 sec at 5–15 Hz pacing frequencies (incremented stepwise by 1 Hz). There was a 30-sec interval after each pacing sequence before beginning the next.

*Electrical measurements*. AV conduction was measured as the time difference between activation recorded by each electrode (right atrium and ventricular apex) during sinus rhythm (Figure 1A,B), as determined by the maximum signal derivative. For the dose–response analysis, we measured the AV delay during sinus rhythm after 15 min exposure to control or BPA-containing media. The sinus heart rate remained stable during perfusion with control media (4.1 ± 1 Hz) but slowed after exposure to 50 and 100 μM BPA (3.6 ± 1 Hz and 3.3 ± 1 Hz, respectively). However, this slowing did not reach significance until the onset of the AV block. All electrical signals were amplified using a Dagan EX4-400 differential amplifier (Dagan Corporation, Minneapolis, MN) and recorded using a PowerLab data acquisition system (ADInstruments, Colorado Springs, CO). Electrical signals, including AV delay and ECG segments, were analyzed using LabChart software (ADInstruments; *n* ≥ 4 signals per concentration for each independent experiment).

*Epicardial conduction velocity (CV) measurements*. We examined the effect of BPA on CV using epicardial electrodes and optical mapping. CV is rate dependent and decreases at high pacing frequencies; such rate-dependent changes are referred to as “CV restitution” (Kleber and Rudy 2004; Qu et al. 2004). Therefore, we implemented a pacing protocol that gradually increased pacing frequencies (5–15 Hz) to reveal the effect of BPA on CV. Epicardial conduction time (seconds) was computed as the difference between the onset of the pacing pulse and the activation time measured from the apical recording electrode (Figure 1A). Epicardial CV (centimeters per second) was computed by dividing conduction time by electrode separation distance. CVs were calculated for each pacing frequency (5–15 Hz). Measurements were normalized to corresponding controls at 5-Hz pacing frequency (near the intrinsic rate). Electrical signals were analyzed using LabChart software (*n* ≥ 4 signals per concentration for each independent experiment).

*Optical mapping of wavefront propagation*. Optical mapping is a powerful technique for studying cardiac electrical conduction, which has been used for more than a decade to reveal mechanisms of arrhythmia (Laughner et al. 2012). We used optical mapping of a potentiometric dye (RH237; Life Technologies, Carlsbad, CA) to study alterations in electrical conduction using techniques developed in our laboratory (Asfour et al. 2011; Kay et al. 2006; Swift et al. 2008). RH237 dye kinetics are faster than changes in transmembrane potential during an action potential (Rosenbaum and Jalife 2001), and RH237 has been used successfully in our laboratory to study cardiac electrical activity. We administered RH237 to the aorta (5 mL of a 10-μM solution) before beginning a pacing protocol (described above). To record optical action potentials, the epicardium was illuminated using an LED spotlight (530/35 nM; Mightex, Pleasanton, CA). RH237 fluorescence was longpass filtered at 680 nm and imaged using a CCD (charge-coupled device) camera (Ixon DV860; Andor Technology, Belfast, UK), as described previously by Mercader et al. (2012). At each pacing frequency, optical signals were acquired and mapping data was quickly viewed to verify continuous elliptical wavefront propagation originating from the pacing electrode. Activation times were identified for each pixel, and local CVs were computed (Bayly et al. 1998) to generate isochronal maps. We computed an average CV by averaging the values of individual CV vectors across the epicardial field of view. Optical action potentials were analyzed using custom Matlab software (MathWorks, Natick, MA) to measure action potential duration at 90% (APD_90_) and depolarization and repolarization times.

*Statistical analysis*. All values are presented as mean ± SE, unless otherwise noted, with *p* < 0.05 considered statistically significant. Mean values are expressed as a percentage of baseline during perfusion with control media before the addition of BPA. The lowest dose showing a statistically different effect (*p* < 0.05) was determined according to one-way or two-way analysis of variance (ANOVA) followed by paired *t*-tests (Bokkers and Slob 2005). All results were computed from a minimum of three independent experiments (animals) for each BPA dose examined (Table 1).

**Table 1:** Summary of statistical significance for each measured electrophysiological parameter.

Measurement	0.1 μM BPA (*n* = 3)	1.0 μM BPA (*n* = 4)	10 μM BPA (*n* = 4)	25 μM BPA (*n* = 7)	50 μM BPA (*n*= 7)	100 μM BPA (*n* = 7)	Potential primary mechanisms of result
Sinus rate slowing	N	N	N	N	Y	Y	Reduced Ca^2+^ current in SA node cells
AV delay	N	N	Y	Y	Y	Y	Reduced Ca^2+^ current in AV node cells
Prolonged PR segment	Y	Y	Y	Y	Y	Y	Impaired conduction in atria, AV node, and bundle branches
Reduced ventricular CV	Y	Y	Y	Y	Y	Y	Reduced Na^+^ current and gap junction conductance
Prolonged APD	N	Y	Y	Y	Y	Y	Reduced K^+^ and Ca^2+^ currents during repolarization
Reduced maximum paced frequency	N	N	N	Y	Y	Y	Increased ventricular refractoriness due to long APD
Abbreviations: Ca^2+^, calcium ion; K^+^, potassium ion; Na^+^, sodium ion. Y denotes significant changes (*p* < 0.05) in parameter differences from control/untreated, and N denotes insignificant changes. Potential primary mechanisms for the effect of BPA on measured parameter differences are shown in the right-hand column. BPA dose ranges correspond to clinical levels in blood (0.1–1 μM) and in urine (0.1–10 μM), as well as to concentrations typically used in toxicological studies (0.1–100 μM). *n* indicates the number of independent experiments/animals examined is shown for each dose.							

In addition, one-way ANOVA was performed to ensure that significant differences in cardiac conduction (including maximum pacing frequency and conduction velocity) were not present between control animals during control media perfusion (*p* > 0.05; data not shown).

## Results

*Effect of BPA exposure on AV conduction delay*. We measured AV conduction delay throughout sinus rhythm, during both control and BPA exposures. AV conduction delay remained stable during control perfusion but lengthened significantly after BPA exposure, beginning at 10 μM (Figure 1B,C). For example, AV conduction increased by 7.5% and 26% after exposure to 10 μM and 25 μM BPA, respectively. Exposure to 100 μM BPA resulted in sustained and complete AV block (see Supplemental Material, Figure S1). Therefore, at 100 μM BPA, conduction delays were computed immediately before the onset of AV block, which usually happened within minutes. We confirmed AV conduction delays by measuring the PR segment time in the ECG, which lengthened after 15 min of exposure to BPA at all concentrations. For example, PR segment time increased by 8% for 0.1 μM BPA and 14% for 10 μM BPA (Figure 1D).

*Heart block after exposure to high BPA concentrations*. Exposure to 100 μM BPA resulted in third-degree AV block. This was detected by dropped ventricular beats, in which atrium impulses failed to propagate to the ventricles (see Supplemental Material, Figure S1). Ventricular activation then resumed and was driven by an accessory pacemaker; changes in QRS morphology and lengthening were also detected in ECG recordings.

**Figure 2:**
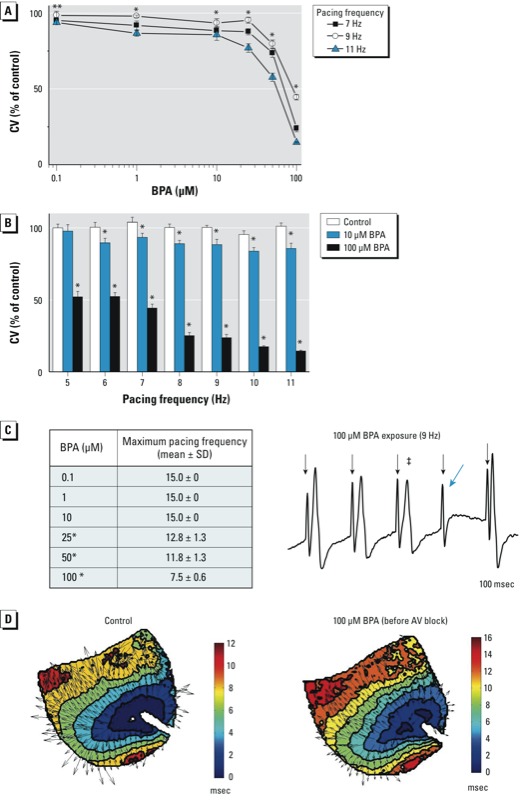
BPA exposure and reduced ventricular CV. (*A*) Reduction in CV at high pacing frequencies (7–11 Hz) 15 min after exposure to BPA (0.1–100 μM). (*B*) Reduction in CV with increasing pacing frequency after exposure to 10 or 100 μM BPA. (*C*) Maximum rate of ventricular activation 15 min after exposure to BPA (left), and example for CV calculation using the last captured signal (denoted by ‡) (right); pacing spikes are indicated by down arrows (↓), and the blue arrow indicates loss of capture. (*D*) Wavefront propagation across the ventricular epicardium at 9 Hz pacing frequency after exposure to control media (left) or 100 μM BPA for 1 min (right); arrows indicate local CVs. Values shown in (*A–C*) are mean ± SE; *n* ≥ 3 for each measurement. *p < 0.05. **p < 0.05 at 9 and 11 Hz, but not 7 Hz. p‑Values for dose response were determined by one- or two-way ANOVA; the lowest dose with statistical significance was determined by paired *t*-tests.

*Effect of BPA exposure on ventricular CV*. Two-way ANOVA indicated significant reductions in CV with both pacing frequency and BPA exposure, compared with control (*p* < 0.0001). The interaction of BPA and pacing frequency was also significant (*p* < 0.001), indicating that BPA altered the CV restitution properties of the tissue. At lower BPA concentrations (0.1–50 μM), significantly reduced CVs were observed at high pacing rates (Figure 2A). For example, while pacing at 11 Hz, the CV for 0.1 μM BPA was 94% of control. For 10 μM BPA, the CV was 94% of control at 7 Hz, and dropped to 86% at 11 Hz. We observed no significant reduction in CV between pacing frequencies during control perfusion (Figure 2B). In contrast, CVs were significantly reduced during BPA exposure, even at pacing frequencies near the intrinsic heart rate (Figure 2B). Each stimulus pulse for pacing rates from 5 to 15 Hz initiated a beat (1:1 capture) during purfusion with control media and 0.1–10 μM BPA. However, the maximum frequency of 1:1 capture was reduced with exposure to higher BPA concentrations (25–100 μM) (Figure 2C, left), indicating an increased refractory period. In these instances, CV was calculated using the last captured signal from a train of successfully propagated impulses (Figure 2C, right). Importantly, we did not detect significance in maximum pacing frequency or CV measurements between animals during control perfusion (ANOVA, *p >* 0.05; data not shown).

Optical mapping data confirmed that paced beats propagated as elliptical wavefronts that emanated from the pacing electrode, even at high BPA concentrations. An example of this is shown in Figure 2D, which illustrates reduction in CV after only 1 min exposure to 100 μM BPA. Averaging the lengths of individual CV vectors across the epicardial field of view revealed a reduction of ventricular CV by 21% across the epicardial surface.

**Figure 3:**
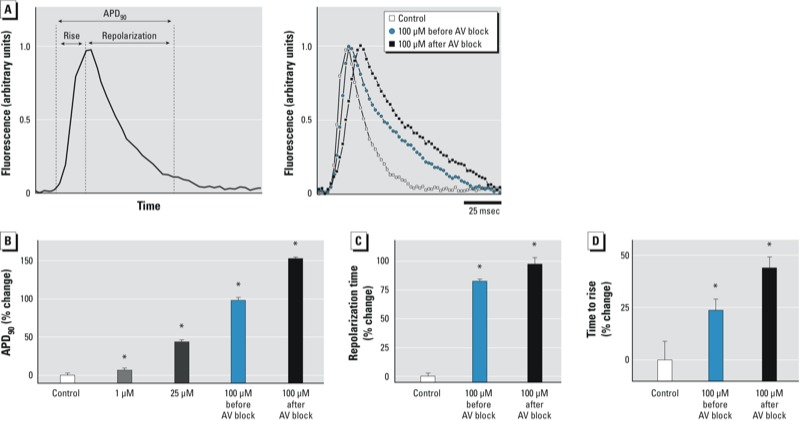
BPA exposure and prolonged ventricular APD_90_. (*A*) Optical action potentials from ventricular tissue (8 Hz); (left) how mearurements were made, and (right) examples of optical action potentials. (*B*) APD_90_ was prolonged after BPA exposure, likely due to longer repolarization (*C*) and depolarization (*D*) times. Dose response determined by one-way ANOVA; lowest dose with significance was determined by paired t-tests. Values shown are mean ± SE; *n* ≥ 3 for each measurement. *p < 0.05. *p*‑Values for dose response were determined by one-way ANOVA; the lowest dose with statistical significance was determined by paired *t*-tests.

*BPA exposure and prolonged ventricular APD*. Exposure to increasing BPA concentrations resulted in slowed CV in a rate-dependent manner, suggesting alterations in the kinetics of membrane depolarization and/or repolarization. Time intervals of depolarization and repolarization were measured using optical action potentials (Figure 3A). We found that APD_90_ was significantly prolonged in hearts exposed to 1–100 μM BPA, in a concentration-dependent manner (Figure 3B). For example, APD_90_ increased by 7% for 1 μM BPA and 44% for 25 μM BPA. Results from 100 μM BPA revealed a larger relative change in repolarization time (98% after third-degree AV block; Figure 3C) than depolarization time (44% after third-degree AV block; Figure 3D).

## Discussion

Although toxic effects of BPA on reproduction and development have been reported (Vandenberg et al. 2007), a link between BPA exposure and cardiovascular disease was found more recently (Lang et al. 2008; Lind and Lind 2011; Melzer et al. 2010, 2012; Shankar et al. 2012). Only a few studies have examined BPA’s adverse cardiac effects and none have described the adverse effects of BPA on electrical conduction within intact hearts. In a study using isolated atrial preparations, Pant et al. (2011) observed that exposure to high concentrations of BPA decreased heart rate via a nitric oxide–dependent signaling mechanism. In another study using isolated ventricular myocytes, Yan et al. (2011) found that BPA exposure (0.001–1 nM), coupled with 17β-estradiol treatment, modified calcium handling.

Our experiments were designed to show the dose–response relationship of BPA on whole-heart conduction abnormalities in female rats. We observed changes in cardiac conduction beginning at 0.1 μM BPA. This concentration is within the range of previously reported human urinary concentrations (0.024–8.5 μM; Table 1) and is within the upper limit of measured human serum levels (0.001–0.3 μM) in high-risk populations (Calafat et al. 2009; Kaddar et al. 2009; Lee et al. 2008; Padmanabhan et al. 2008; Schönfelder et al. 2002; Vandenberg et al. 2010; Wang et al. 2012). Such concentrations may be present in individuals chronically exposed to high levels of BPA (i.e., industrial workers) and those with reduced metabolic capacities (i.e., fetuses and infants). Although BPA is not considered a persistent compound, there is controversy regarding whether or not BPA is immediately cleared from the body (Teeguarden et al. 2012; vom Saal et al. 2012). Importantly, in the present study, we observed changes in cardiac conduction after acute (≤ 15 min) BPA exposure, a time frame that is significantly less than BPA’s reported half-life (Shin et al. 2004; Volkel et al. 2002).

In the present study, we observed significant, although modest, changes in cardiac conduction beginning at 0.1 μM BPA. Notably, small changes in ion channel expression and/or electrical conduction can lead to pathological outcomes (Amin et al. 2010; Grant 2009). At low micromolar concentrations of BPA (10 μM), we observed a concentration-dependent slowing of AV conduction that began within 15 min of exposure (Table 1). AV conduction delay was confirmed by measuring PR segment time, with delays found at BPA doses as low as 0.1 μM BPA. We also found that BPA exposure resulted in prolonged APD_90_ and slowed ventricular CV beginning at 1 μM and 0.1 μM, respectively. CV is rate dependent and is reduced at high pacing frequencies, a phenomenon known as CV restitution that is primarily established by the recovery kinetics of sodium channels (Kleber and Rudy 2004; Qu et al. 2004). We observed CV slowing at high pacing rates in control measurements, as well as a significantly greater slowing of CV after BPA exposure. High pacing frequencies mimic increased heart rates, which could result from increased work/exercise or stress. When heart rates are elevated, individuals with increased BPA exposure could be susceptible to adverse cardiac effects, including electrical conduction that is slower than normal. In our study we also observed that the maximum heart rate that could be achieved was significantly reduced after BPA exposure. For example, at 25 μM BPA, maximum pacing frequency was reduced from 15 Hz to 12.8 Hz. This result indicates an increased refractory period, which could be caused by prolonged sodium channel inactivation resulting from reduced potassium current and longer action potential duration (Jalife et al. 2009).

The cardiac effects of BPA were pronounced at high concentrations. Exposure to 100 μM BPA resulted in complete AV block and QRS interval widening, which is consistent with slowed ventricular conduction that may be attributed to reduced gap junction conductance (Gillum et al. 2009), reduced sodium channel activity, and activations that originate outside the specialized conduction system. We also detected a lengthening in APD_90_, with a larger relative change in repolarization time than in depolarization time. This indicates that BPA-induced tissue refractoriness could be the dominant mechanism of conduction failure at high concentrations and pacing rates. We recognize that cardiac exposure to 100 μM BPA exceeds a clinically relevant exposure concentration; however, these observed effects may provide a hint to the underlying mechanisms of BPA on the conduction system, which can be examined further, and also allow for direct comparison with previously reported mechanical effects (Pant et al. 2011).

Our results are significant because altered electrical conduction is a mechanism of reentrant arrhythmias (Roden 1996), which can cause tachycardia and fibrillation in both the atria and ventricles. The effects of BPA exposure—even at low BPA concentrations—that we observed in the present study, could be symptomatic at high heart rates, particularly in elderly patients whose hearts tend to be larger and more fibrotic (Biernacka and Frangogiannis 2011). In addition, action potential prolongation is a common mechanism of conduction block, particularly at high heart rates (rate-dependent block), and is known to be a trigger for reentrant arrhythmias (Rosenbaum et al. 1973). Reduced CV may also cause reentrant arrhythmias, particularly in patients with dilated hearts (Akar et al. 2004). These adverse effects are important, even though we did not observe such arrhythmias in our studies, which is most likely the result of the small size of the rat heart and its intrinsically low susceptibility to arrhythmias.

The present study compliments previous studies of the effect of BPA on mechanical function. For example, two studies reported that BPA-induced mechanical dysfunction was sex-specific because of BPA’s estrogenic properties (Belcher et al. 2012; Yan et al. 2011). In our study, as in these previous studies, each animal served as its own control to minimize any potential effects of estrous cyclicity. This reduced the influence of the estrous cycle on measurements of cardiac conduction, as evidenced by a lack of statistical significance in measurements between animals during control perfusion (ANOVA, *p* > 0.05; data not shown). As a result, in our paired-animal controlled study using excised hearts, we observed slowing of cardiac electrical conduction after BPA exposure.

**Figure 4:**
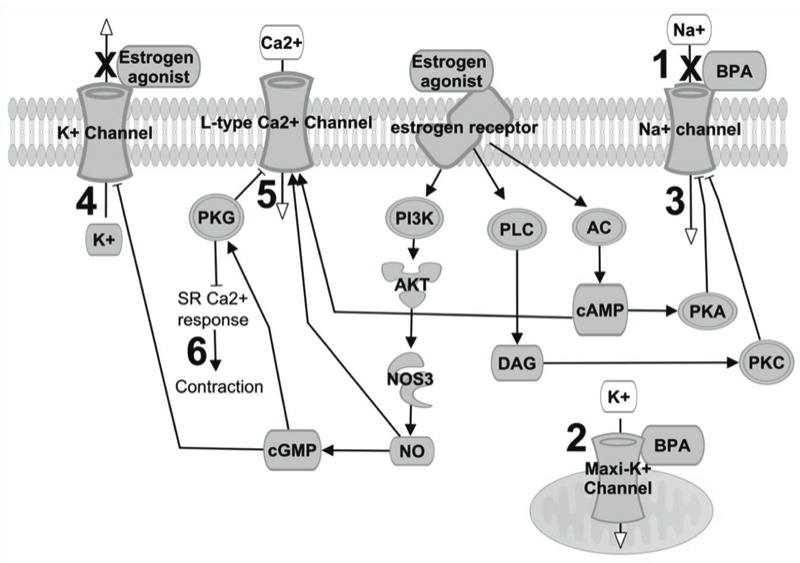
Possible mechanisms underlying BPA’s impairment of cardiac conduction. Abbreviations: AC, adenylate cyclase; AKT, protein kinase B; Ca^2+^, calcium; cAMP, cyclic adenosine monophosphate; cGMP, cyclic guanosine monophosphate; DAG, diacylglycerol; ER, estrogen receptor; K^+^, potassium; maxi-K^+^ channels, Ca^2+^-activated K^+^ channels; Na^+^, sodium; NO, nitric oxide; NOS3, nitric oxide synthase 3; PI3K, phosphoinositide 3-kinase; PKA, protein kinase A; PKC, protein kinase C; PKG, protein kinase G; PLC, phospholipase C. BPA binding can (*1*) block voltage-gated Na^+^ channels or (*2*) activate maxi-K^+^ channels (located in the mitochondria of cardiomyocytes, or in sarcolemma of cardiac neurons and endothelial cells). ER agonists can (*3*) inhibit Na^+^ current via activation of the PKC–PKA pathway, (*4*) inhibit the K^+^ current, and (*5*) inhibit the L-type Ca^2+^ current via the NO/cGMP/PKG pathway, which acts as an antagonist to cAMP activation. This effect is concentration dependent because NO can activate Ca^2+^ channels at basal concentrations. (*6*) BPA can reduce cardiac contractility, an effect that is also dependent on NO concentration (see Supplemental Material, Table S1).

Such impaired electrical conduction may potentially be attributed to BPA’s interaction with ion channels and/or estrogen receptors (Figure 4; see also Supplemental Material, Table S1). BPA can bind directly to and block the Nav1.5 sodium channel (O’Reilly et al. 2012), which is responsible for phase 0 depolarization in ventricular myocytes (Figure 4, mechanism 1). Inhibition of the fast sodium current by BPA would certainly reduce ventricular CV. BPA can also activate Maxi-K channels in coronary smooth muscle (mechanism 2; Asano et al. 2010). A similar interaction with sarcolemma potassium channels could hyperpolarize cardiomyocytes and decrease cardiac excitability. Modifications in either sodium or potassium channel current could also explain the increased tissue refractoriness we observed at high pacing frequencies.

Because BPA is classified as a xenoestrogen, it is plausible that the impaired cardiac conduction we observed was mediated by its interaction with estrogen receptors (ERs) and the resultant downstream pathways. Animals that undergo ovariectomy have shortened PR intervals and shorter ventricular refractory periods (Saba et al. 2002), which can be explained by the effects of ER agonists on multiple ion currents. ER agonists can inhibit voltage-gated sodium current (mechanism 3; Wang et al. 2013) and decrease potassium current, which can prolong APD and repolarization time (mechanism 4; Berger et al. 1997; Kurokawa et al. 2008; Nakajima et al. 1999; Tanabe et al. 1999). ER agonists can also decrease L-type calcium current (mechanism 5; Jiang et al. 1992; Meyer et al. 1998; Nakajima et al. 1999), which can prolong ventricular APD (Berger et al. 1997; Tanabe et al. 1999). Because the L-type calcium current is the primary depolarizing current in sinoatrial and AV nodal cells, a reduction in calcium current can lead to AV block (Hancox and Mitcheson 1997; Kawai et al. 1981). Similar to other ER agonists (Jiang et al. 1992; Liew et al. 2004), BPA has also been found to decrease cardiac contractility, possibly through its interaction with ERs and/or activation of the nitric oxide/cGMP (cyclic guanosine monophosphate) pathway (mechanism 6; Belcher et al. 2012; Pant et al. 2011). An increase in nitric oxide levels can also attenuate the L-type calcium current (Han et al. 1997), which could be an important mechanism for dropped beats and AV block at high BPA concentrations.

## Conclusion

Our results show that acute BPA exposure alters electrical conduction in excised hearts from female rats. This could be the result of a block in the Nav1.5 sodium channel, a reduction in gap junction conductance, a reduction in calcium channel opening due to a nitric oxide/cGMP pathway, and/or inhibition of potassium channels via the estrogenic properties of BPA. Because of the complex interactions between these pathways (Figure 4), additional studies are needed to fully identify the mechanisms responsible for BPA’s effects, and to determine whether these mechanisms differ by exposure levels and treatment time.

One limitation of the present study is that we examined the effects of BPA on cardiac conduction after acute *ex vivo* exposure. Whether such conditions are relevant to chronic *in vivo* exposure is not yet known and requires additional studies. These additional experiments are important because BPA may motivate conduction abnormalities in individuals with preexisting heart conditions, such as AV conduction dysfunction, disease of the cardiac electrical conduction system, or fibrosis of the atria and/or ventricles. Other high-risk populations, such as industrial workers, prenatal and neonatal patients with reduced metabolic capacity, and elderly patients with substantial cardiac fibrosis may also be affected. Overall, our findings emphasize the importance of examining how BPA exposure affects heart electrophysiology and determining whether chronic *in vivo* exposure can cause or exacerbate conduction abnormalities in patients with preexisting heart conditions and in other high-risk populations.

## Supplemental Material

(291 KB) PDF
