# Supplementary material for: Bisphenol A Exposure and Cardiac Electrical Conduction in Excised Rat Hearts
Source: Environ Health Perspect. 2014 Jan 31;122(4):384–90. doi: 10.1289/ehp.1206157 (PMC3984226; doi:10.1289/ehp.1206157)
Supplement: (291 KB) PDF [file ehp.1206157.s001.pdf]

Supplemental Material

Bisphenol A Exposure and Cardiac Electrical Conduction in Excised Rat Hearts

Nikki Gillum Posnack, Rafael Jaimes III, Huda Asfour, Luther M. Swift, Anastasia M. Wengrowski, Narine Sarvazyan, and Matthew W. Kay

Table of Contents

Figure S1 Page 2

Table S1 Page 4–5

References Page 6–7

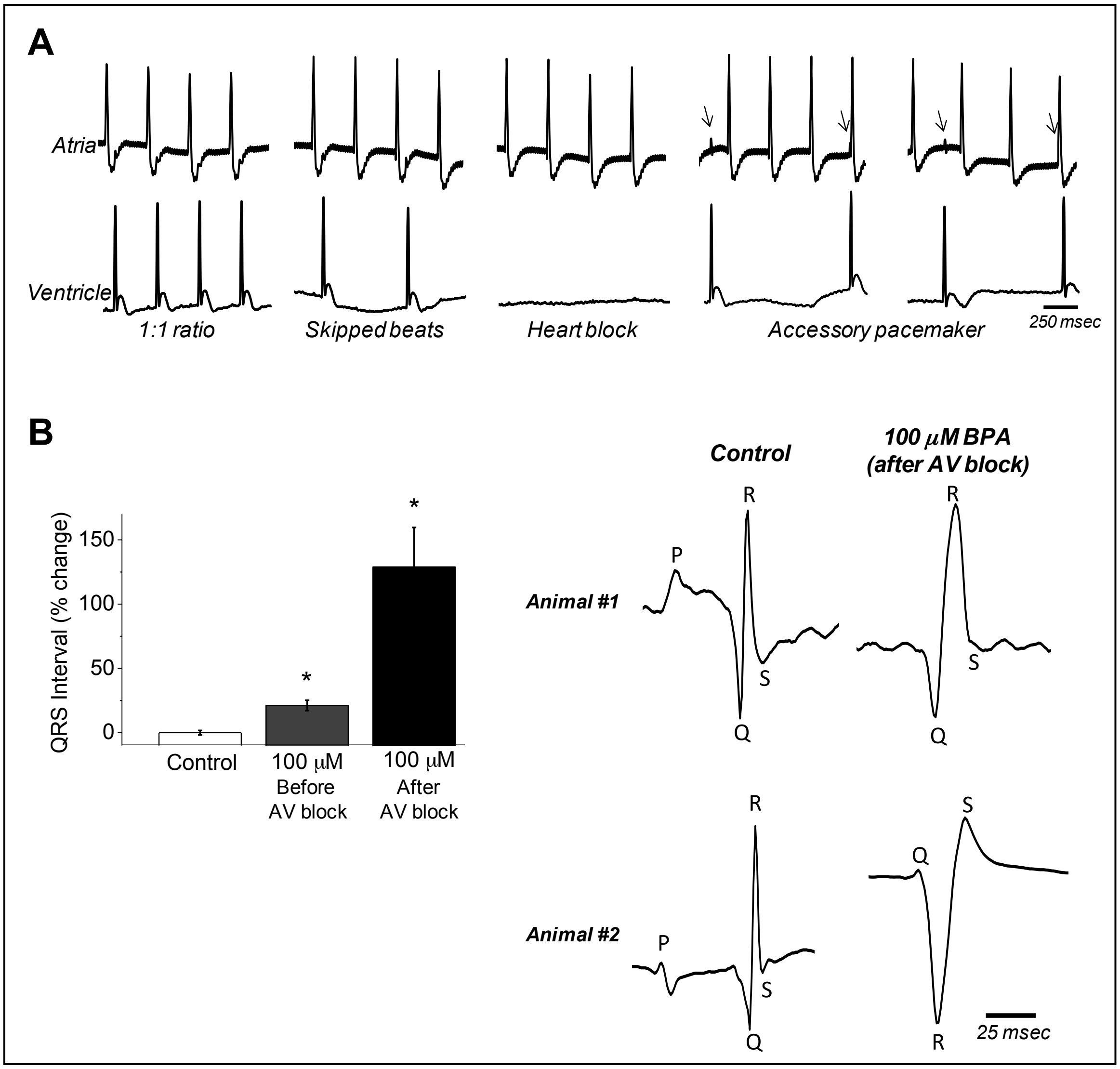

Figure S1. Exposure to high concentrations of BPA results in 3rd degree AV block. **A.** Time course of AV block after exposure to 100 μM BPA (atrial and ventricular signals are shown). AV conduction ratio progressed from 1:1 to 2:1 (2nd degree AV block). Across all studies, 2nd degree AV block occurred within 3.6 ± 2.4 minutes after raising BPA concentration to 100 μM. After this, hearts quickly progressed to complete failure of AV conduction (3rd degree AV block). Across all studies, 3rd degree AV block occurred within 4.3 ± 2.6 min after raising BPA to 100 μM. At this point, there was a short pause where no electrical activity was measured with the apical recording electrode. Ventricular activation then resumed and was driven by an

accessory pacemaker that activated the ventricles independently of the atrium (denoted by arrows [Zipes and Jalife, 2009]). **B.** Left: 100 μM BPA increases the QRS interval ($n \geq 3$, $*p < 0.05$). Right: ECG signals show widening of the QRS interval and changes in QRS morphology.

Table S1. Experimental studies that investigate possible mechanisms underlying BPA's impairment of conduction velocity (illustrated in Figure 4).

Possible mechanisms	Source	Chemical	Cell type	Experimental outcome
1	O'Reilly et al. 2012	Bisphenol A	HEK293 ($Nav_{1.5}$ transfected)	BPA binds and blocks voltage-gated Na^+ channel. Effect at 1 μM BPA, acute treatment (4 min)
2	Asano et al. 2010	Bisphenol A	Coronary muscle neurons	BPA binds and activates Maxi-K^+ channels. Effect at 10 μM BPA, acute treatment (< 1 min, reversible)
3	Liu et al. 2010	Raloxifene	Ventricular cardiomyocytes	Raloxifene (ER agonist) decreased Na^+ current
3	Wang et al. 2013	Bisphenol A	Dorasal root ganglion neurons	BPA inhibits tetrodotoxin-sensitive and resistant Na^+ current. BPA's effect is blocked by PKC and PKA inhibitors. IC50 = 11.6 μM BPA (5 min), partially reversible with washout.
4	Druzin et al. 2011	Estradiol	Medial preoptic neurons	17-β-estradiol (ER agonist) binds to open K^+ channels to reduce voltage-gated K^+ current
4	Moller and Netzer 2006	Estradiol	CHO cells	17-β-estradiol inhibited voltage-gated K^+ channels
4	Kurokawa et al. 2008	Estradiol	Ventricular cardiomyocytes, CHO cells (hERG transfected), Guinea pig hearts	17-β-estradiol suppressed K^+ current (hERG channel gating), results in QT prolongation
4	Tanabe et al. 1999	Estradiol	Ventricular cardiomyocytes	17-β-estradiol prolonged APD and repolarization time mainly by inhibiting K^+ current
4	Berger et al. 1997	Estradiol	Ventricular cardiomyocytes	17-β-estradiol reduced K^+ current and prolonged APD
4	Nakajima et al. 1999	Estradiol	Atrial cardiomyocytes	17-β-estradiol inhibited K^+ current
5	Lee et al. 2002	Estradiol	Dorsal root ganglion neurons	17-b-estradiol inhibits L-type Ca^{2+} current via activation of pertussis toxin-sensitive G-proteins
5	Tanabe et al. 1999	Estradiol	Ventricular cardiomyocytes	17-β-estradiol reduced L-type Ca^{2+} current, prolonged APD and repolarization time
5	Berger et al. 1997	Estradiol	Ventricular cardiomyocytes	17-β-estradiol reduced L-type Ca^{2+} current
5	Nakajima et al. 1999	Estradiol	Atrial cardiomyocytes	17-β-estradiol inhibited cAMP-enhanced L-type Ca^{2+} current, effect was blocked by cGMP or L-NAME (NOS inhibitor) pretreatment

Possible mechanisms	Source	Chemical	Cell type	Experimental outcome
5	Jiang et al. 1992	Estradiol	Ventricular cardiomyocyte	17-β-estradiol decreased peak inward Ca^{2+} current
5	Meyer et al. 1998	Estradiol	Ventricular cardiomyocytes	17-β-estradiol inhibited L-type Ca^{2+} current
6	Jiang et al. 1992	Estradiol	Ventricular cardiomyocyte	17-β-estradiol has a negative inotropic effect, decreases myocyte cell shortening
6	Pant et al. 2011	Bisphenol A	Isolated atrial preparations	BPA decreased atrial contractility, effect was blocked by pretreatment with L-NAME (NOS inhibitor) or methylene blue (guanylyl cyclase inhibitor). Effect at 0.1 μM BPA (10 min)
6	Belcher et al. 2011	Bisphenol A	Ventricular cardiomyocytes	BPA reduced contractility via estrogen receptor beta signaling, pretreatment with L-NAME (NOS inhibitor) did not alter BPA's effects. Effect at 10^{-12}M BPA, acute treatment (2-7min)
6	Liew et al. 2004	Raloxifene	Ventricular cardiomyocytes	Raloxifene decreased cell shortening, decreased Ca^{2+} transient amplitude, and decreased L-type Ca^{2+} current. Raloxifene effects inhibited by pretreatment with estrogen receptor antagonist

References

Asano S, et al. 2010. Bisphenol A activates maxi-K (K(ca)1.1) channels in coronary smooth muscle. Br J Pharmacol 160(1):160-170.

Belcher SM, Chen Y, Yan S, Wang HS. 2011. Rapid estrogen receptor-mediated mechanisms determine the sexually dimorphic sensitivity of ventricular myocytes to 17beta-estradiol and the environmental endocrine disruptor bisphenol A Endocrinology 153(2):712-20.

Berger F, Borchard U, Hafner D, Putz I, Weis TM. 1997. Effects of 17beta-estradiol on action potentials and ionic currents in male rat ventricular myocytes. Naunyn Schmiedebergs Arch Pharmacol 356(6):788-796.

Druzin M, Malinina E, Grimsholm O, Johansson S. 2011. Mechanism of estradiol-induced block of voltage-gated K+ currents in rat medial preoptic neurons. PLoS One 6(5):e20213.

Jiang C, Poole-Wilson PA, Sarrel PM, Mochizuki S, Collins P, MacLeod KT. 1992. Effect of 17 beta-oestradiol on contraction, Ca2+ current and intracellular free Ca2+ in guinea-pig isolated cardiac myocytes. Br J Pharmacol 106(3):739-745.

Kurokawa J, Tamagawa M, Harada N, Honda S, Bai CX, Nakaya H et al. 2008. Acute effects of oestrogen on the guinea pig and human IKr channels and drug-induced prolongation of cardiac repolarization. J Physiol 586(Pt 12):2961-2973.

Lee DY, Chai YG, Lee EB, Kim KW, Nah SY, Oh TH et al. 2002. 17Beta-estradiol inhibits high-voltage-activated calcium channel currents in rat sensory neurons via a non-genomic mechanism. Life Sci 70(17):2047-2059.

Liew R, Stagg MA, MacLeod KT, Collins P. 2004. Raloxifene acutely suppresses ventricular myocyte contractility through inhibition of the L-type calcium current. Br J Pharmacol 142(1):89-96.

Liu H, Yang L, Jin MW, Sun HY, Huang Y, Li GR. 2010. The selective estrogen receptor modulator raloxifene inhibits cardiac delayed rectifier potassium currents and voltage-gated sodium current without QTc interval prolongation. Pharmacol Res 62(5):384-390.

Meyer R, Linz KW, Surges R, Meinardus S, Vees J, Hoffmann A et al. 1998. Rapid modulation of L-type calcium current by acutely applied oestrogens in isolated cardiac myocytes from human, guinea-pig and rat. Exp Physiol 83(3):305-321.

Moller C, Netzer R. 2006. Effects of estradiol on cardiac ion channel currents. Eur J Pharmacol 532(1-2):44-49.

Nakajima T, Iwasawa K, Oonuma H, Morita T, Goto A, Wang Y et al. 1999. Antiarrhythmic effect and its underlying ionic mechanism of 17beta-estradiol in cardiac myocytes. Br J Pharmacol 127(2):429-440.

O'Reilly AO, Eberhardt E, Weidner C, Alzheimer C, Wallace BA, Lampert A. 2012. Bisphenol a binds to the local anesthetic receptor site to block the human cardiac sodium channel. PLoS One 7(7):e41667.

Pant J, Ranjan P, Deshpande SB. 2011. Bisphenol A decreases atrial contractility involving NO-dependent G-cyclase signaling pathway. J Appl Toxicol 31(7):698-702.

Tanabe S, Hata T, Hiraoka M. 1999. Effects of estrogen on action potential and membrane currents in guinea pig ventricular myocytes. Am J Physiol 277(2 Pt 2):H826-33.

Wang Q, Cao J, Hu F, Lu R, Wang J, Ding H et al. 2013. Effects of estradiol on voltage-gated sodium channels in mouse dorsal root ganglion neurons. Brain Res 246(5):391-7.

Zipes DP, Jalife J. 2013. Cardiac electrophysiology: from cell to bedside. Philadelphia, PA: Saunders, an imprint of Elsevier Inc.
